# Photocatalytic optimization of ZnO–Ga_2_O_3_ composite thin films for PEC water splitting: effects of thickness, environment, and annealing temperature

**DOI:** 10.1039/d5ra03463a

**Published:** 2025-08-04

**Authors:** Yerbolat Tezekbay, Tolagay Duisebayev, Zhamilya Taubaldiyeva, Alshyn Abduvalov, Nurxat Nuraje, Olzat Toktarbaiuly

**Affiliations:** a Renewable Energy Laboratory, National Laboratory Astana (NLA), Nazarbayev University Astana 010000 Kazakhstan olzat.toktarbaiuly@nu.edu.kz; b Department of Chemical and Materials Engineering, School of Engineering and Digital Sciences, Nazarbayev University Astana 010000 Kazakhstan; c Department of Physics, School of Sciences and Humanities, Nazarbayev University Astana 010000 Kazakhstan alshyn.abduvalov@nu.edu.kz

## Abstract

This study reports a systematic investigation into the photoelectrochemical (PEC) performance of Ga_2_O_3_/ZnO (GZO) composite thin films fabricated *via* RF magnetron sputtering. GZO films were deposited on FTO/Glass and titanium (Ti) foil substrates, with key fabrication parameters – namely deposition time, annealing gas atmosphere, and annealing temperature – systematically varied to optimize photocatalytic activity. Surface morphology and crystallinity were evaluated using SEM and XRD, respectively, revealing that both deposition time and annealing conditions significantly influence grain structure and crystallinity, which in turn affect PEC performance. Among the tested conditions, films deposited for 25 minutes and annealed in air exhibited optimal performance, with annealing at 600 °C on Ti foil substrates yielding the highest photocurrent density of 1.7 × 10^−4^ A cm^−2^ at 1.23 V *vs.* RHE. Electrochemical impedance spectroscopy (EIS) confirmed improved charge transfer properties at this temperature, although stability testing indicated potential trade-offs between performance and long-term durability. These findings highlight the critical role of thermal and atmospheric control during post-deposition treatment in tailoring the structural and electronic properties of GZO thin films. The optimized GZO photoanodes demonstrate strong potential for low-cost, efficient, and scalable solar hydrogen production, contributing to the advancement of sustainable energy technologies.

## Abstract 2

Optimized ZnO–Ga_2_O_3_ thin films for PEC water splitting by tuning thickness, annealing, and environment, enhancing photocurrent density and revealing key structure–performance links for scalable solar hydrogen production.

## Graphical abstract

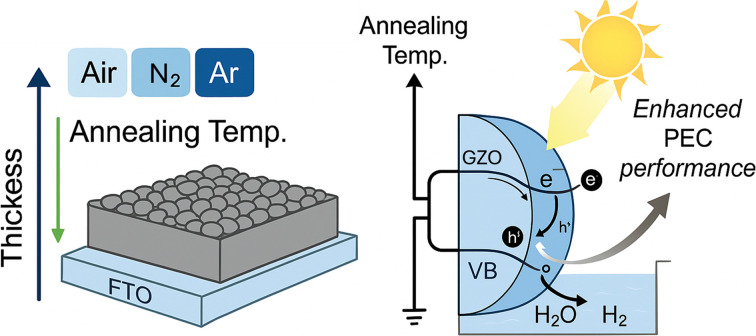

## 1. Introduction

The increasing consumption of organic fuels has significantly escalated the global carbon footprint, adversely affecting the planet's ecology.^1^ This scenario has driven the urgent need to explore alternative energy sources, such as solar, wind, hydrothermal, and hydrogen energy. Among the various methods of hydrogen production, including methane pyrolysis,^2^ water electrolysis,^3^ and water vapor interaction with coal,^4^ green hydrogen production stands out due to its minimal carbon footprint. A promising avenue within green hydrogen energy is photoelectrochemical (PEC) water splitting.^5^

PEC water splitting involves the decomposition of water into hydrogen and oxygen at electrodes under solar irradiation. Over the past decade, 89 studies on PEC water splitting have been documented in the Web of Science, demonstrating its growing prominence. A key advantage of PEC lies in its direct utilization of solar energy without intermediate conversion steps. Essential requirements for PEC cells include appropriate band gap materials, efficient carrier separation, chemical stability, low cost, and environmental sustainability.^6^

Water splitting requires a minimum energy input of 1.23 eV, necessitating the use of wide band gap materials such as TiO_2_, ZnO, WO_3_, and MgO.^7–10^ The efficiency of PEC systems can be enhanced through the use of conductive and transparent solar substrate materials like indium tin oxide (ITO) and fluorine-doped tin oxide (FTO), widely applied in solar panels, displays, and optoelectronic devices.^11–13^

Modern electrode materials for PEC applications include graphene-based compounds, MXenes, polymers, metal oxides and their multilayered composites.^14–18^ Among these, metal oxides have exhibited superior performance, with ZnO emerging as a particularly promising candidate due to its wide band gap (3.37 eV), non-toxic nature, and cost-effectiveness, making it one of the potential candidates for photocatalysis.^19^ The introduction of dopants such as Ga, Cu, In, and Al into ZnO has been shown to enhance its electrical and optical properties, thereby improving PEC cell efficiency.^20–23^ Overall, there are plenty of materials reported with water splitting usage potential.^24,25^

Various synthesis methods are employed for fabricating PEC electrode materials, including hydrothermal synthesis, sol–gel processes, atomic layer deposition (ALD), chemical vapor deposition (CVD), and magnetron sputtering.^24–28^ Each method offers unique advantages,^29,30^ contributing to the optimization of PEC systems for sustainable hydrogen production. Amid composites, ref. 31 reports GaO/ZnO thin films obtained by sol–gel method varying GaO concentration up to 3%.

This study investigates the photoelectrochemical (PEC) activity of ZnO–Ga_2_O_3_ composite (GZO) thin films prepared *via* RF-magnetron sputtering at various deposition times, resulting in films with different thicknesses, which was not studied before. The novelty of this work lies in the systematic examination of the relationship between film thickness, annealing conditions, and PEC performance. Results indicate that optimizing surface morphology through structural modifications in GZO films significantly enhances PEC activity. These findings provide a foundation for advancing PEC water-splitting technologies by leveraging controlled deposition and annealing processes.

## 2. Materials and methods

### 2.1 GZO preparation

GZO thin films were deposited on fluorine-doped tin oxide (FTO) substrates and titanium (Ti) foil using a Kurt J. Lesker radio frequency (RF) magnetron sputtering system. A ZnO/Ga_2_O_3_ target (95 wt% ZnO, 5 wt% Ga_2_O_3_) with a purity of 99.998% was obtained from Kurt J. Lesker. The sputtering target measured 2.00′′ in diameter, 0.250′′ in thickness, and had a density of 4.19 g cm^−3^.

Prior to loading into the sputtering system, the FTO substrates were sequentially cleaned using an ultrasonic bath with acetone and methanol for 20 minutes each. The thickness of the transparent FTO substrates was measured using a Dektak XT (Bruker) profilometer, yielding a value of 350 nm.

The deposition chamber was initially evacuated to a base pressure of 1 × 10^−7^ Torr, and a working pressure of 5 mTorr was maintained during deposition with pure argon (99.99%) introduced at a flow rate of 5 sccm as the working gas. The GZO films were deposited at room temperature with deposition times of 10, 20, and 30 minutes. The RF sputtering power was set at 30 W, with the substrate holder rotating at 81.6° and a speed of 10 rpm to ensure film uniformity. After deposition, the thin films were thermally annealed in air at 500 °C for 30 minutes to improve crystallinity.

All furnace annealing experiments were conducted at 10 °C min^−1^ temperature rise rate and holding 20 °C with natural cooling.

### 2.2 PEC analysis testing

The PEC (photoelectrochemical) activity of the GZO thin films was evaluated using a three-electrode setup, comprising GZO as the working electrode, Ag/AgCl as the reference electrode, and a platinum wire as the counter electrode. Measurements were conducted in 0.1 M Na_2_SO_4_ aqueous solution as the electrolyte under solar illumination provided by a Newport LCS-100 solar simulator (AM 1.5, 100 W), calibrated to 1 sun using a reference silicon cell. Linear sweep voltammetry (LSV) and chronoamperometry (CA) measurements were performed using a PalmSens4 potentiostat, with potential values (V *vs.* Ag/AgCl) converted to the RHE scale using the equation:

1 *E*_RHE_ = *E*_Ag/AgCl_ + 0.059 pH + 0.205

here, 0.205 V is the standard potential of the Ag/AgCl electrode at 25 °C.

## 3. Results and discussion

Optimization experiments for GZO thin films were initially carried out by adjusting the film thickness through controlled deposition times. This was followed by testing different gas atmospheres, and finally, by varying the annealing temperature based on the outcomes of the previous optimizations. All these experiments were initially performed on FTO/Glass substrates. However, the final set of temperature variation tests was also repeated using Ti-foil substrates, as FTO/glass cannot tolerate high-temperature annealing.

**Fig. 1:**
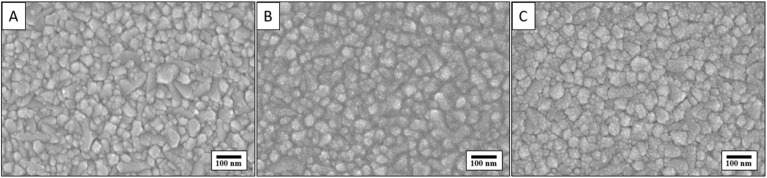
SEM images of GZO thin films deposited by increasing sputtering time: (A) 10 min, (B) 20 min, (C) 30 min.

The SEM images in Fig. 1 show the surface morphology of GZO composite thin films deposited on FTO glass substrates for 10 (A), 20 (B), and 30 minutes (C). At 10 minutes (A), the film exhibits larger, irregularly shaped grains with noticeable porosity and uneven coverage, influenced by the inherently rough and textured nature of the FTO substrate, which hinders uniform initial nucleation. By 20 minutes (B), the film becomes more uniform and compact with finer grains, indicating enhanced surface coverage and nucleation density that better align to the FTO surface, resulting in changed morphology than in 10 min deposited sample. But still, we see the effect of FTO substrate on thin film morphology at his stage. At 30 minutes (C), grain growth is evident, leading to slightly coarser grains and more pronounced boundaries, although the film remains continuous. Overall, the evolution from A to C highlights how increased deposition time improves film uniformity and coverage of GZO thin films, with 25 minutes appearing optimal before grain coarsening begins to dominate. The roughness of the underlying FTO plays a significant role in early-stage nucleation, influencing film continuity and grain distribution in shorter deposition times.

**Fig. 2:**
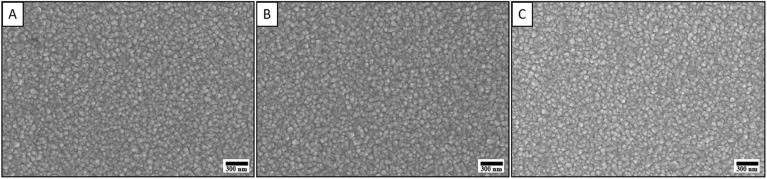
SEM images of GZO thin films deposited in various gas environments: (A) nitrogen, (B) argon, (C) air.

Fig. 2 shows the surface SEM images of GZO thin films deposited on FTO/Glass substrates in various gas environments including nitrogen (N_2_), argon (Ar) and air fixing deposition time at 25 min. SEM images show that the films have a uniform and homogeneous surface with a granular structure where the grain sizes are similar for all samples and no significant morphological differences are observed between the films annealed under different gas conditions. This observation suggests that the annealing environment, whether inert (Ar), reducing (N_2_), or oxidizing (Air), does not significantly affect the surface morphology of the GZO films. The granular texture and uniform grain distribution indicate that the growth mechanism and crystallization process remain stable under these annealing conditions.

Additionally, we preformed cross-sectional SEM imaging on sample deposited for 25 min after annealing in air as shown in Fig. S1. The average thickness was estimated as 75 nm for 25 min deposition, consequently, since deposition conditions were same for all samples, average deposition rate is 3 nm min^−1^: 10 min – 30 nm, 20 min – 60 nm and 30 min – 90 nm.

Fig. S2 shows surface SEM images of samples on FTO/Glass substrates annealed by increasing annealing temperature from 300 °C to 550 °C. It was revealed that varying annealing temperature will not have influence on surface morphology. Since, FTO/Glass substrate has limit in increasing temperature further, Ti foil substrates were used for next experiments for temperature increasing.

**Fig. 3:**
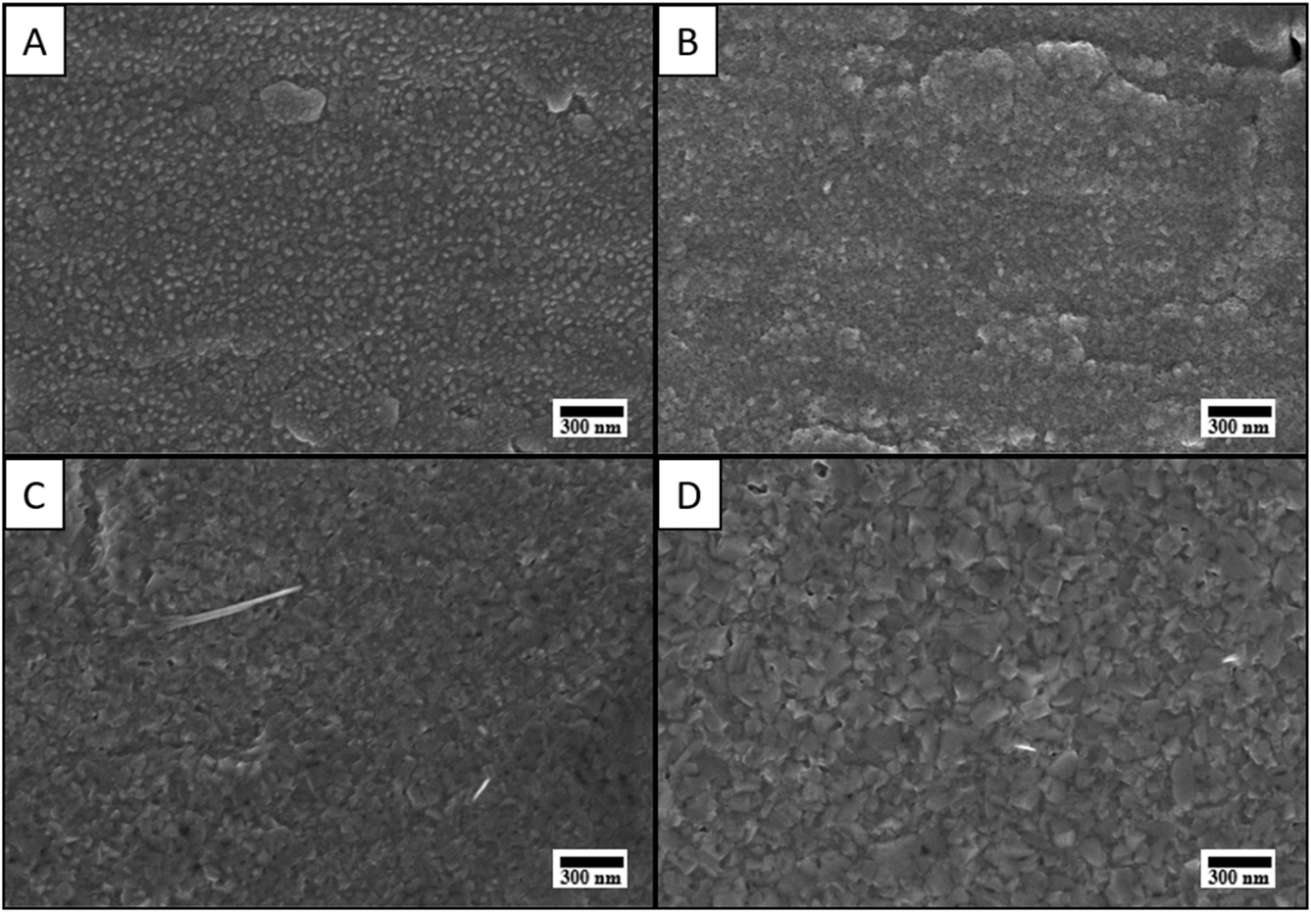
Surface SEM images of GZO thin films deposited on Ti foil by increasing annealing temperature: (A) 400 °C, (B) 500 °C, (C) 600 °C and (D) 650 °C.

Fig. 3 exhibits surface SEM images of GZO thin films on Ti foil substrates annealed by increasing temperature from 400 °C to 600 °C. As the surface of Ti foil substrates has rough morphology than FTO/Glass substrates, obtained GZO thin films follow same microstructure as substrate by holding its grain like morphological properties at 400 °C and 500 °C of annealing. However, at 600 °C the GZO thin film shows distinct changes in comparison to 500 °C annealed sample. The surface is characterized by a dense, less granular structure composed of uniformly distributed sharply edged hills with well-defined boundaries. The hills exhibit a polyhedral or rounded morphology, suggesting significant grain growth and crystallization due to high-temperature annealing. There is no visible porosity or cracking. The elevated annealing temperature has likely promoted grain coalescence leading to the observed compact and tightly packed structure. At 650C, the GZO thin films surface becomes more dense, less granular with distinct and increased uniformly distributed sharply edges. At some points holes or porous points are seen.

**Fig. 4:**
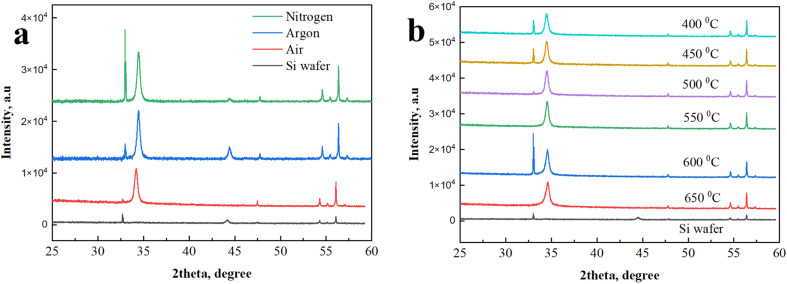
XRD patterns of GZO thin films on Si wafer: (a) annealed in various gas environments and (b) annealed at various temperatures from 400 °C to 650 °C.

Fig. 4 shows XRD patterns of samples annealed on Si wafer varying gas environment and at different temperatures up to 650 °C. It was done to check crystalline structures of samples on various optimization conditions. The choice of Si wafer was because XRD signal from samples on FTO/Glass substrates was not seen due to very thin nature of the GZO films and overlapping FTO peaks. Fig. S3 confirms that XRD signal on FTO substrates are not seen due to high intense and overlapping FTO peaks. XRD results on Si wafer reveal that on varying gas environments and temperature crystalline structure of GZO film was not changed. Peak appeared at 34.4° stays in all samples for all modifications and it corresponds to the hexagonal wurtzite phase (002),^32^ (JCPDS 77-0191).

**Fig. 5:**
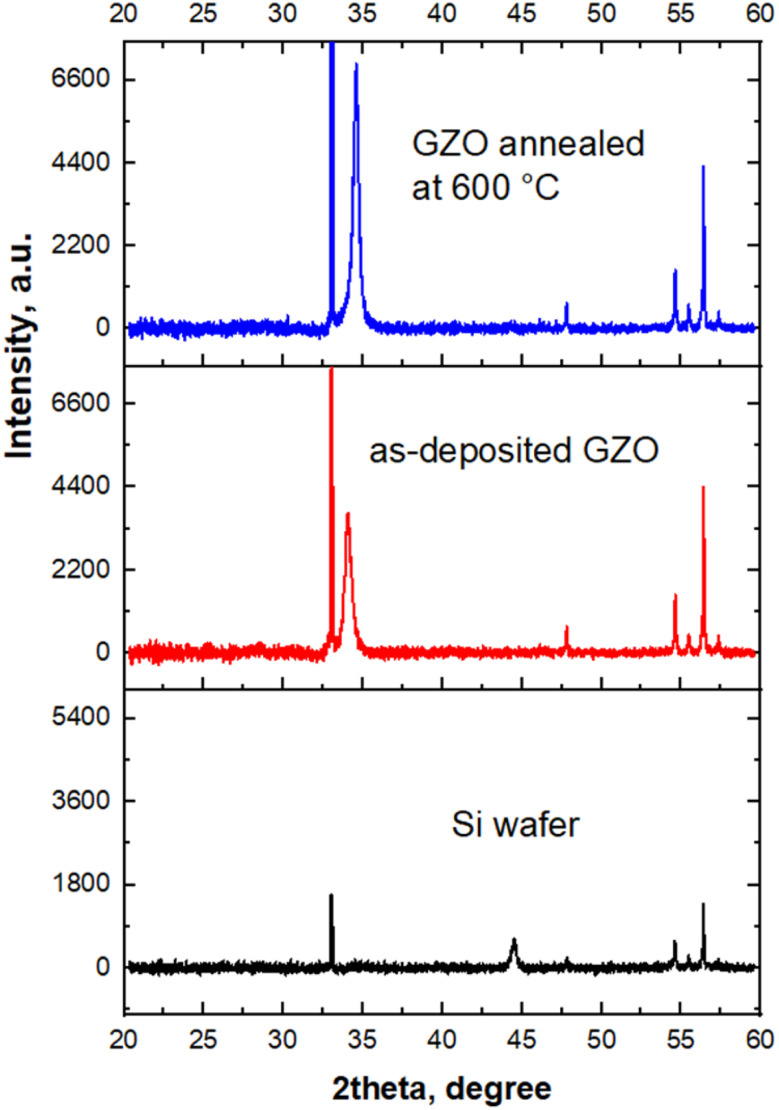
XRD patterns of as-deposited and annealed at 600 °C GZO thin films on Si wafer.

It was revealed that peak at 34.4° appears also in as-deposited sample as-shown in Fig. 5 meaning its crystalline nature from sputter deposition step at room temperature. Our results show that structural properties of as-deposited sample were further enhanced. This fact was confirmed by enhanced intensity of XRD peak of sample annealed at 600 °C in same intensity scale relative to as-deposited sample, seen in Fig. 5.

The next, PEC properties of samples were examined using LSV, EIS, and current–time measurements under simulated solar irradiance with chopper. Optimization experiments of GZO thin films were done first, varying thin films thickness by controlling deposition time, then using various gas environments, lastly, varying annealing temperature depending on previous optimization results. All optimization experiments were conducted on FTO/Glass substrates, however the last experiment related to temperature variation was repeated using Ti-foil substrates, since FTO/Glass substrates cannot withstand high temperature annealing experiments.

**Fig. 6:**
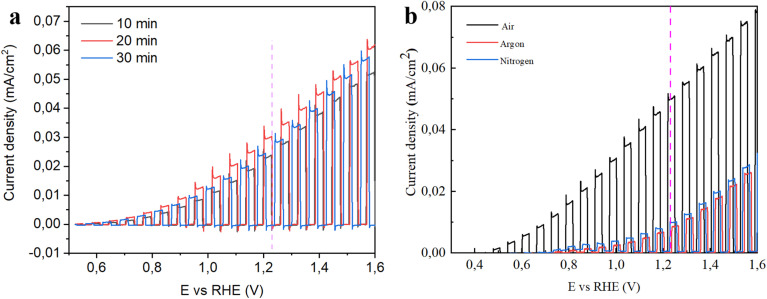
Chopped LSV curves of GZO samples (a) deposited at various deposition times and (b) annealed at 500 °C in air, nitrogen and argon environments after deposition.

Fig. 6a shows LSV curves for GZO samples deposited at various time periods: 10 min – 30 nm, 20 min – 60 nm, and 30 min – 90 nm. Thickness optimization of GZO thin films for PEC applications plays a critical role in determining the overall efficiency of photoanodes. An appropriately tuned thickness ensures a balance between sufficient light absorption and efficient charge carrier transport. GZO thin films that are too thin may not absorb enough incident photons, resulting in reduced generation of electron–hole pairs and consequently lower photocurrent densities. On the other hand, excessively thick films can hinder charge carrier transport due to increased recombination losses arising from longer charge diffusion paths. Therefore, optimizing the film thickness is essential to maximize light harvesting while minimizing charge recombination. As we observe on our results, thickness variation has less effect on overall photocurrent generation ability for magnetron sputtering GZO films than other preparation parameters. It was revealed that overall photocurrent density change is not huge for thickness variation. Photocurrent generation increases from 10 min (2.4 × 10^−5^ A cm^−2^) deposited sample to 20 min (3.02 × 10^−5^ A cm^−2^) deposited sample and decreases at 30 min (2.7 × 10^−5^ A cm^−2^) deposited samples at 1.23 V *vs.* RHE scale. Therefore, for the next optimization steps we chose 25 min deposition as optimal thin films deposition parameter.

Fig. 6b shows chopped LSV curves for the samples annealed in various gas environments holding same annealing time, and temperature *etc.* As a result of photocurrent generation at 1.23 V *vs.* RHE scale show (8.1 × 10^−6^ A cm^−2^ for argon, 1 × 10^−5^ A cm^−2^ for nitrogen and 5 × 10^−5^ A cm^−2^ for Air environments) annealing in different gas environments significantly affects the properties of GZO composite thin films, thereby influencing their performance. The best performed sample was annealed in air. In inert atmospheres such as argon and nitrogen, oxygen diffusion is limited, which can result in higher concentrations of oxygen deficiencies and structural disorders. While a certain level of oxygen vacancies may enhance conductivity, excessive defects often act as recombination centers for photogenerated charge carriers, thereby reducing PEC efficiency. In contrast, annealing in air provides an oxygen-rich environment that promotes better crystallinity and facilitates the passivation of oxygen vacancies. This leads to reduced charge recombination, improved carrier lifetime, and more efficient charge separation and transfer. In this study, the films annealed in air exhibited the highest photocurrent density, indicating superior PEC activity. The enhanced performance in air is attributed to the improved structural quality and favorable electronic properties induced by the oxidative annealing atmosphere.

**Fig. 7:**
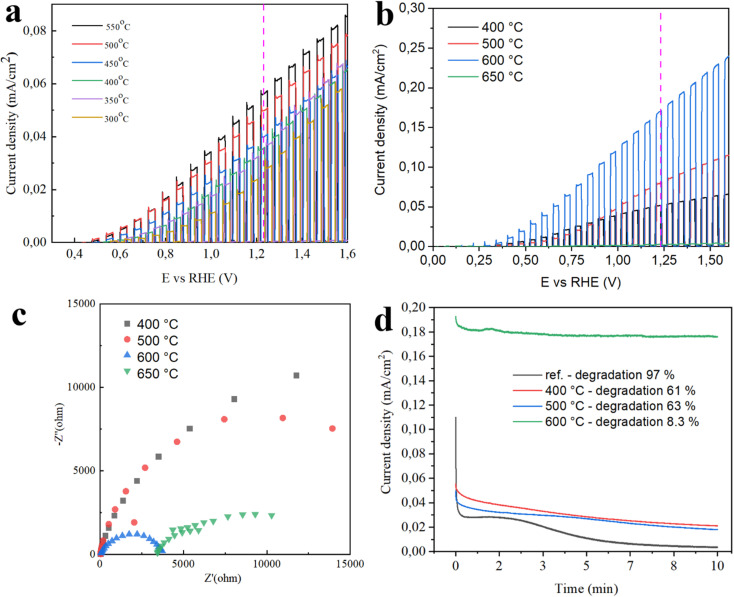
Chopped LSV curves of GZO samples annealed at various temperatures (a) on FTO, (b) on Ti foil, and (c) EIS curves (Nyquist plot) on Ti foil, (d) current–time measurements for 10 min fixing potential at 0.69 V *vs.* Ag/AgCl ref electrode.

To determine the optimal annealing temperature for maximizing photocurrent, GZO thin films were deposited onto FTO substrates and annealed in an air atmosphere at various temperatures, specifically 300 °C, 350 °C, 400 °C, 450 °C, 500 °C, and 550 °C (Fig. 7a). At lower temperatures, incomplete crystallization and higher defect densities can lead to poor charge carrier mobility and increased recombination losses. As the annealing temperature increases, the crystallinity of the film generally improves, promoting better charge transport and reducing defect-related recombination. However, excessively high temperatures can lead to grain coarsening, interdiffusion, or stress-induced cracking, which may degrade PEC performance. Therefore, identifying an optimal annealing temperature is essential to achieving a balance between improved structural quality and minimal defect states. In the case of GZO films, our results in Fig. 7a show that annealing at 550 °C was found to yield the best PEC response on FTO substrates, likely due to enhanced crystallinity and more efficient charge separation and transport at this temperature. However, the maximum annealing temperature was limited to 550 °C to avoid exceeding the melting point of the FTO substrate. To further study, we performed annealing at different temperatures on Ti foil substrates that can withstand high temperatures.

Fig. 7b shows chopped LSV curves of GZO thin films annealed at 400 °C, 500 °C, 600 °C and 650 °C. The highest performance of photoanode was observed on sample annealed at 600 °C, showing 1.7 × 10^−4^ A cm^−2^ photocurrent generation at 1.23 RHE scale. As was explained, annealing at 650 °C shows deterioration of the sample leading to the poor performance on photoactivity. It might be due to grain coarsening, interdiffusion, or stress-induced cracking that happens at excessive annealing temperatures. Additionally, high temperatures can promote interdiffusion between Ga and Zn species, potentially resulting in the formation of undesirable secondary phases or disrupting the intended stoichiometry and band alignment of the composite thin film. Furthermore, thermal expansion mismatch between the film and the substrate at high temperatures can introduce mechanical stress, which may lead to microcrack formation or delamination. These structural degradations negatively impact charge transport pathways and increase carrier recombination, ultimately diminishing the PEC performance of the thin films. Additionally, factors such as composite interface and active reactive sites play an important role in photoactivity of the obtained materials. In our case, since GZO thin films have the same interface composition varying only by thickness, temperature, and with the gas environment. Its interface with electrolyte stays similar for all thin films. However, SEM images of varying thickness gave change on morphology that leads to the change on reactive sites. The better reactive site for photocatalytic enhancement is seen on sample with the deposition time of 25 min. The mechanism lies in the increase of active area at this thickness that shows the highest chemical interaction between the material and electrolyte.

EIS results in Fig. 7c confirm the fact that sample annealed at 650 °C on Ti foil has improved charge–transport properties. It can be attributed to enhanced charge transfer characteristics at the electrode–electrolyte interface. At this elevated temperature, further crystallization and grain growth likely occurred, which can reduce grain boundary resistance and improve overall film conductivity. These changes contribute to lower charge transfer resistance (*R*__ct_), as seen in the Nyquist plot, indicating more efficient separation and transport of photogenerated carriers.

Stability testing for 10 min in Fig. 7d shows that GZO thin films annealed at 600 °C were higher stable (with degradation of 8.3%) than those reference and annealed at 400 °C, 500 °C. This is likely because the high temperature of 600 °C causes less stress, microcracks, or surface damage in the film, which makes it degrade less during PEC operation. In contrast, reference samples gave the worst stability with degradation of 97%.

**Fig. 8:**
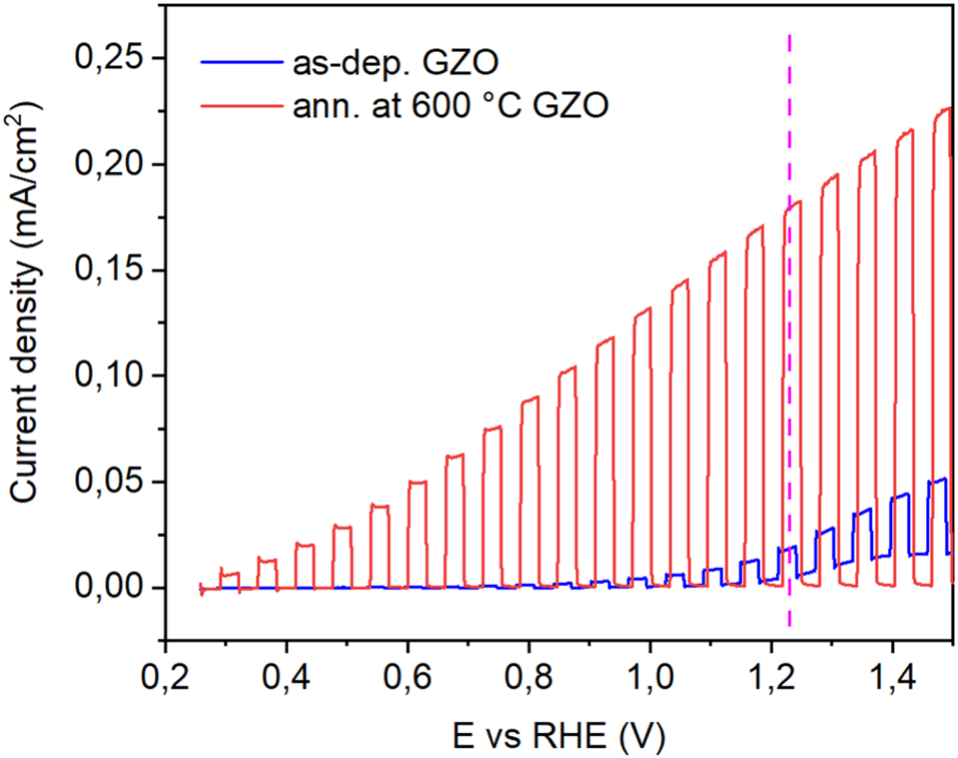
Chopped LSV curves of as-deposited and annealed at 600 °C GZO samples.

At the end of optimization experiments, we performed a comparison of the best performed sample GZO on Ti foil with as-deposited GZO. Fig. 8 shows chopped LSV curves of GZO thin film annealed at 600 °C relative to as-deposited. The optimized best sample shows 10 times better performance than as-deposited sample by increasing photocurrent generation from 1.8 × 10^−5^ A cm^−2^ to 1.8 × 10^−4^ A cm^−2^ at 1.23 RHE scale.

To confirm the presence of elements in this work XPS survey measurement of one sample was carried out. Fig. S4 shows that samples used in experiments indeed have presence of Ga, Zn, O and C elements.

Optimization experiments in our report does not include UV-vis with Tauc plots and Mott–Schottky measurements of samples on Ti foil substrates due to their opaque nature, however previous studies confirm the n-type nature of both ZnO and Ga_2_O_3_, and their composite formation has been shown to enhance band structure modulation and charge separation, key factors for efficient PEC performance.^33^

## 4. Conclusion

This work report optimization of GaO-ZnO (GZO) composite thin films for photocatalytic applications on FTO/Glass and Ti foil substrates. The study underscores the importance of annealing conditions including gas environment, thickness and annealing temperature. Comprehensive characterization revealed that as-deposited sample has crystalline nature and annealing enhances crystallinity directly influencing PEC performance. GZO films deposited on Ti foil and annealed at 600 °C achieved the highest photocurrent with improved charge carrier dynamics and light absorption properties.

This study provides a pathway for developing low-cost, efficient, and scalable materials for green hydrogen production *via* PEC water splitting. The insights gained lay a foundation for further advancements in sustainable energy technologies, emphasizing the potential of GZO thin films in addressing global energy challenges.

## Conflicts of interest

The authors declare that they have no known competing financial interests or personal relationships that could have appeared to influence the work reported in this paper.

## Supplementary Material

RA-015-D5RA03463A-s001

## Data Availability

The data supporting this article have been included as part of the SI. SI figures provide additional structural and compositional data on GZO thin films deposited on FTO glass. See DOI: https://doi.org/10.1039/d5ra03463a.
