# Supplementary material for: Photocatalytic optimization of ZnO–Ga2O3 composite thin films for PEC water splitting: effects of thickness, environment, and annealing temperature
Source: RSC Adv. 2025 Aug 4;15(34):27586–93. doi: 10.1039/d5ra03463a (PMC12320224; doi:10.1039/d5ra03463a)
Supplement: RA-015-D5RA03463A-s001 [file RA-015-D5RA03463A-s001.pdf]

Supplementary Information (SI) for RSC Advances.
This journal is © The Royal Society of Chemistry 2025

Photocatalytic optimization of ZnO-Ga_2O_3 composite thin films for PEC water splitting: effects of thickness, environment, and annealing temperature.

Yerbolat Tezekbay[a], Tolagay Duisebayev[a], Zhamilya Taubaldiyeva[b], Alshyn Abduvalov[c*], Nurxat Nuraje[a,b] and Olzat Toktarbaiuly[a*]

[a]*Renewable Energy Laboratory, National Laboratory Astana (NLA), Nazarbayev University, Astana 010000, Kazakhstan*
[b]*Department of Chemical and Materials Engineering, School of Engineering and Digital Sciences, Nazarbayev University, Astana 010000, Kazakhstan*
[c]*Department of Physics, School of Sciences and Humanities, Nazarbayev University, Astana 010000, Kazakhstan*

Supporting information

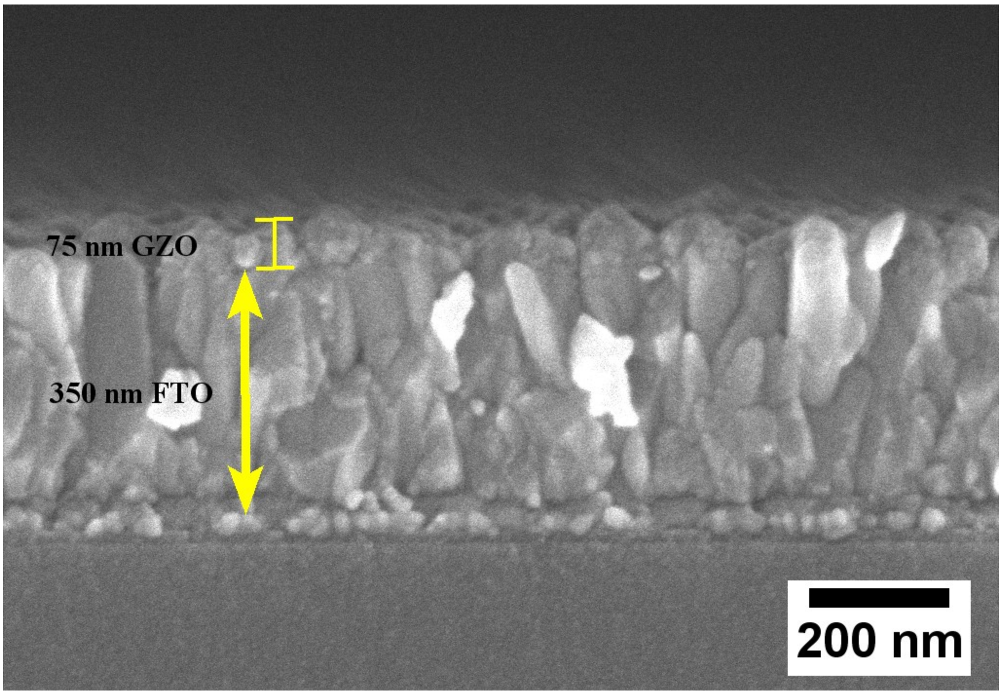

Fig. S-1 Cross-sectional SEM image of GZO sample on FTO/Glass deposited at 25 min.

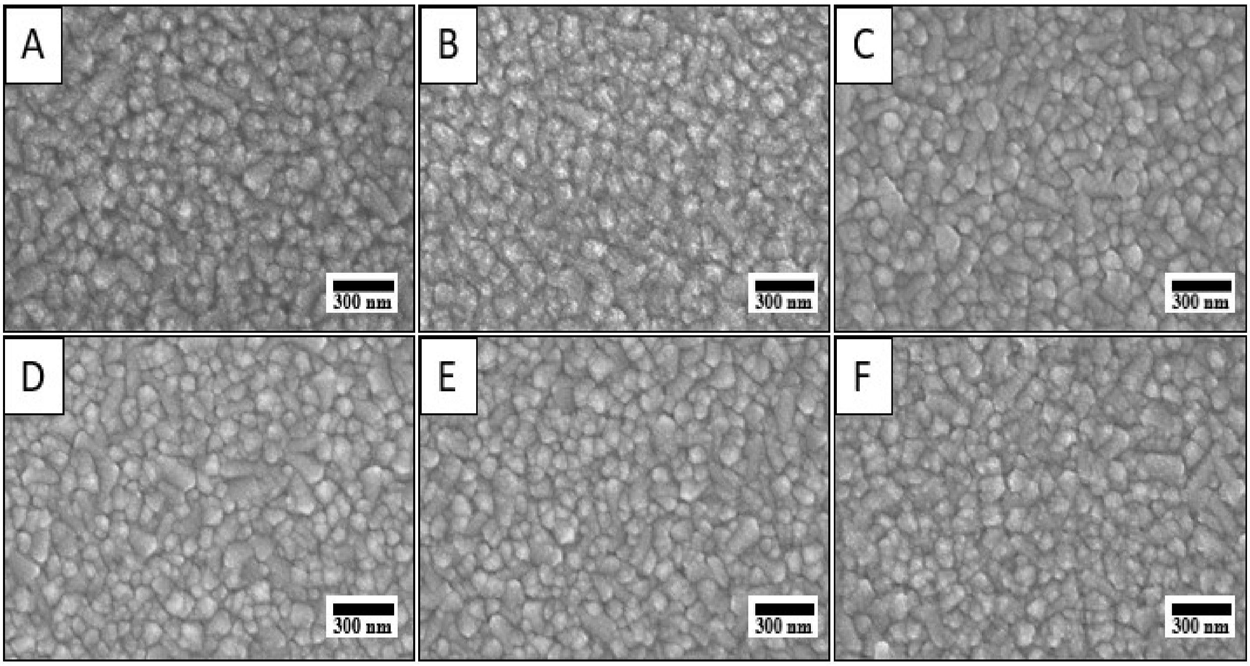

Fig. S-2 Surface SEM images of the GZO thin films deposited on FTO glass substrates by annealing in air at temperatures: (a) 300°C, (b) 350°C, (c) 400°C, (d) 450°C, (e) 500°C, and (f) 550°C.

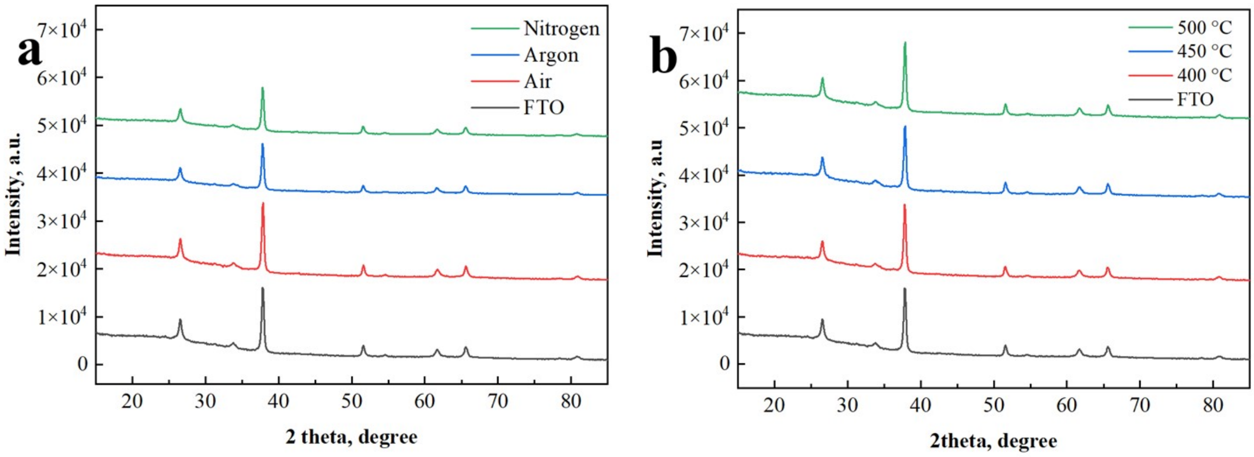

Fig. S-3 XRD patterns of the GZO thin films deposited on FTO glass substrates and annealed (a) in various gas environments, (b) at various temperatures. All patterns show only FTO peaks.

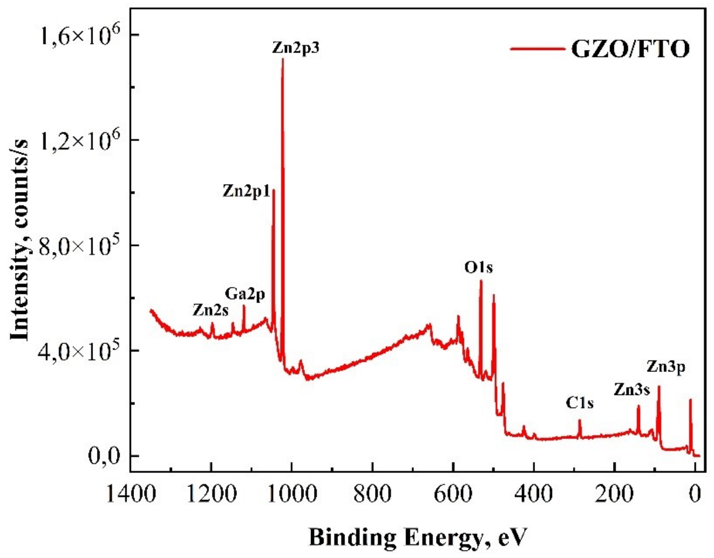

Fig. S-4 Survey XPS of the 25 min. deposited GZO thin films deposited on FTO glass substrates and annealed.
